# Different Postharvest Responses of Fresh-Cut Sweet Peppers Related to Quality and Antioxidant and Phenylalanine Ammonia Lyase Activities during Exposure to Light-Emitting Diode Treatments

**DOI:** 10.3390/foods8090359

**Published:** 2019-08-23

**Authors:** Gludia M. Maroga, Puffy Soundy, Dharini Sivakumar

**Affiliations:** Department of Crop Sciences, Phytochemical Food Network Research Group, Tshwane University of Technology, Pretoria West 0001, South Africa

**Keywords:** photo technology, shelf life, *Capsicum annuum* L., postharvest quality, bioactive compounds, antioxidant activity

## Abstract

The influence of emitting diode (LED) treatments for 8 h per day on functional quality of three types of fresh-cut sweet peppers (yellow, red, and green) were investigated after 3, 7, 11, and 14 days postharvest storage on the market shelf at 7 °C. Red LED light (660 nm, 150 μmol m^−2^ s^−1^) reduced weight loss to commercially acceptable level levels (≤2.0%) in fresh-cuts of yellow and green sweet peppers at 7 and 11 d, respectively. Blue LED light (450 nm, 100 μmol m^−2^ s^−1^) maintained weight loss acceptable for marketing in red fresh-cut sweet peppers up to 11 d. Highest marketability with minimum changes in color difference (∆E) and functional compounds (total phenols, ascorbic acid content, and antioxidant activity) were obtained in yellow and green sweet pepper fresh-cuts exposed to red LED light up to 7 and 11 d, respectively, and for red sweet pepper fresh-cuts exposed to blue LED light for 11 d. Red LED light maintained the highest concentrations of β carotene, chlorophyll, and lycopene in yellow, green, and red sweet pepper fresh-cuts up to 7 d. Similarly, blue LED light showed the highest increase in lycopene concentrations for red sweet pepper fresh-cuts up to 7 d. Red LED (yellow and green sweet peppers) and blue LED (red sweet pepper) lights maintained phenolic compounds by increasing phenylalanine ammonia lyase activity. Thus, the results indicate a new approach to improve functional compounds of different types of fresh-cut sweet pepper.

## 1. Introduction

Light*-*emitting diode (LED) lights are becoming increasingly popular in horticulture because of their energy efficiency, cost effectiveness, long life, nonresidual and nontoxic effects, small size, and low heat production on exposed surfaces [1]. Because of these advantages, the use of LED lighting during storage (cold rooms) or transportation (refrigerated trucks) could be an alternative solution to reduce postharvest losses and maintain product quality and shelf life [2].

The most important aspect of LEDs is the ease to control and maintain a specific monochromatic spectrum, favoring photomorphogenic responses such as growth and synthesis of secondary metabolites in plants [1]. The use of LED light with a high red to far red (R:FR) light ratio was shown to increase lycopene synthesis in tomatoes [3]. White-blue LED lights improved flavonoid and antioxidant activity (FRAP and ABTS^+^) in brussels sprouts (outer leaves) and carotenoid contents in broccoli during postharvest storage [4,5]. Furthermore, the significant effect of LED lights on growth and metabolism of several postharvest pathogens and food contaminants have been proven previously [6]. Therefore, using light manipulation to improve or maintain the antioxidants in postharvest storage is regarded as a chemical-free green energy technology [1].

Sweet or bell peppers (*Capsicum annuum* L., family Solanaceae) are a widely consumed vegetable and provide a rich source of ascorbic acid, flavonoids, phenolic acids, and carotenoids, known as antioxidants, with numerous health benefits [7]. The composition of antioxidants in sweet pepper depends on many factors such as variety, cultivation conditions, the degree of ripeness at harvest, and postharvest handling. Sweet peppers contain high concentrations of total phenols, which decrease as the fruit ripens [8]. Moreover, sweet peppers are produced in different colors such as red, yellow, orange, green, white, and purple, commonly known as less pungent pepper varieties [9].

Minimal or fresh processing of fresh produce is becoming much more common than using the intact product in foodservice and retail markets as a convenience product, as consumer preferences towards ready-to-use or ready-to-eat vegetables are increasing. The fresh-cut processing of sweet peppers consists of a cutting operation, which keeps the plant tissue metabolically active and highly perishable, shortening its shelf life and limiting its marketability [10]. In addition, changes in texture, color, and functional compounds in fresh-cut products occur during storage or marketing. 

To our knowledge, no information is available on the application of LED lights on fresh-cut sweet pepper types to improve its quality parameters and bioactive compounds during postharvest storage (at the market shelf). Our preliminary investigation with increasing LED exposure times affected the sensory quality and weight loss at 7 °C (unpublished data). Therefore, the objective of this study was to investigate the effect of 8 h exposure to red and blue LED lights, primarily (1) on the weight loss, marketability, and color difference; secondly (2) on the retention of lipophilic pigments (chlorophyll in green sweet pepper, β-carotene in yellow sweet pepper, and lycopene in red sweet pepper); thirdly (3) on the accumulation of antioxidants (total phenols, quercetin, and ascorbic acid) and antioxidant activities (FRAP); and finally (4) to improve the understanding of the influence of LED lights on the activity of phenylalanine ammonia lyase (PAL) on the accumulation of phenolic compounds, which may offer a new approach to enhance the antioxidant levels in fresh-cut sweet peppers during display at the market shelf at 7 °C. The PAL enzyme converts phenylalanine to ammonia and trans-cinnamic acid during the first step in the phenylpropanoid pathway [11].

## 2. Materials and Methods

### 2.1. Plant Material and Light-Emitting Diode (LED) Light Treatment

Three sweet pepper cultivars—cv. ‘California Wonder’ (green), ‘King of the North’ (red), and ‘Citrine F1 Hybrid’ (yellow)—were harvested at commercial maturity from a farm that supplies regularly to the Tshwane Fresh Produce Market (Pretoria West, South Africa). The sweet peppers were transported to the laboratory within 3–4 h after harvest. Sweet peppers that were free from decay, defects, or damages were selected and cut into rings (3 cm thick) using a sharp sterile knife and thereafter dipped in 0.1 mL L^−1^ NaOCl (pH 2.5~3) for 5 min. The fresh-cut rings were rinsed with sterile water, and the excess water was removed by blotting with sterile tissue paper. Yellow, red, or green sweet pepper samples of 125 g each were packed separately in black polystyrene trays and wrapped with biaxially oriented polypropylene (BOPP) film without sealing (atmosphere gas composition) in order to reduce the moisture loss. Each type of LED treatment consisted of 20 replicate tray packs per storage time and were placed in a random position in a line. The tray packs were held at 7 °C for 85%relative humidity (RH) for up to 3, 7, 11, and 14 days to simulate cold storage at supermarkets, and samples were withdrawn at designated intervals for the analysis. During cold storage, fresh-cut yellow, red, and green sweet peppers were subjected to either red LED (660 nm, 150 μmol m^−2^ s^−1^) or blue LED (450 nm, 100 μmol m^−2^ s^−1^) light for 8 h per day, based on our previous trials. Exposure to 8 h white light (white cool fluorescent lamps; Phillips, Fluotone 40 W) and continuous darkness for the designated storage time were included as controls. After withdrawing the samples at designated intervals, the 10 replicate treatments for each time range were evaluated for weight loss, overall marketability, and color change (∆E) over time. The other 10 replicates per treatment were snap-frozen in liquid nitrogen and held at −80 °C to determine changes in bioactive compounds and PAL enzyme activity.

### 2.2. Weight Loss

The initial weight of 5 replicate pepper rings was standardized to weigh 100 g on day 0, before the LED storage trials. On each sampling time (3, 7, 11, and 14 days), a standard scale (Milton Keynes, UK) was used to record the weight. The differences in weight between the sampling days were calculated with reference to the initial weight and expressed as percent weight loss.

#### Overall Marketability

A panel of 30 individuals (15 men and 15 women), aged 25–30, who were familiar with sweet pepper and consumed it on a regular basis, assessed the overall marketability of a randomized selection of samples. Ten replicate punnets per treatment were randomly presented in uncovered and unlabeled tray packs. Evaluation was performed on overall marketability, mainly based on the absence of discoloration due to browning, using a 5 point hedonic scale (5 = excellent, absence of browning, marketable at the supermarkets that meet stringent quality standards*;* 4 = good, 5%–10% discoloration, marketable; 3 = average 25% discoloration, limited marketability; 2 = poor, 50%, unmarketable; 1 = 100% unmarketable [12].

### 2.3. Color Change (∆E)

A Minolta CR-400 chromameter (Minolta, Osaka, Japan) calibrated with a standard white tile was used to measure the color values of fresh-cut sweet peppers. Three measurements were recorded per fresh-cut sample. In the CIE color system, a* values describe the intensity of redness (+) and greenness (−), and b* values describe the intensity of yellowness (+) or blueness (−). The L* values describe lightness (black = 0, white = 100). The color changes were quantified in the L* (lightness), color coordinates a*, and b* values using the following formula [13]:

(1) $$\Delta E_{ab}^{*}=\sqrt{{\left(L_{2}^{*}-L_{1}^{*}\right)}^{2}+{\left(a_{2}^{*}-a_{1}^{*}\right)}^{2}+{\left(b_{2}^{*}-b_{1}^{*}\right)}^{2}}$$

### 2.4. Phytochemical Contents

#### 2.4.1. Ascorbic Acid Content

The ascorbic acid content was determined using the 2,6-dichlorophenolindophenol dye titration method [12] for the different samples. These results were expressed as grams of ascorbic acid per kilogram FW (fresh weight).

#### 2.4.2. Chlorophyll Content

The total chlorophyll content was determined from extractions of 50 mg pepper samples in 50 mL methanol [12]. *C*entrifugation of the resulting mixture was performed using a centrifuge (Hermle Labortechnik, Wehingen, Germany) at 6000× *g* for 5 min at 4 °C. The chlorophyll a (Chl a) and chlorophyll b (Chl b) contents were determined by measuring the absorbance of the resulting supernatant at 646 and 662 nm (microplate reader SpectrostarNano, BMG-LABTEC, Ortenberg, Germany). The total chlorophyll content was calculated and expressed as grams of chlorophyll per kilogram FW.

#### 2.4.3. Lycopene and β-Carotene Contents

Lycopene and β-carotene were extracted in 2 mL acetone:*n*-hexane (4:6), as previously described [12], using snap-frozen samples (0.5 g) of red and yellow fresh-cut sweet peppers. After centrifuging at 6000× g for 5 min at 4 °C, the resulting supernatant (200 μL) was used to determine the absorbances at 663, 645, 505, and 453 nm (Microplate Reader, Zenyth 200 rt Biochrom Ltd., Cambridge, UK). The acetone:*n*-hexane (4:6) solvent was used as blank reference. The lycopene and *β*-carotene contents were calculated using the following formulas [14]:

Lycopene [g kg^−1^ FW] = −0.0458*A*663 + 0.204*A*645 + 0.372*A*505 − 0.0806*A*453; (2)

*β*-carotene [g kg^−1^ FW] = 0.216*A*663 − 1.220*A*645 + 0.304*A*505 − 0.452*A*453. (3)

#### 2.4.4. Total Phenolic and Flavonoid Contents

Total phenolic and flavonoid contents were determined, as described previously [12], by homogenizing 1 g sweet pepper fruit pericarp in 10 mL acetone:ethanol (1:1 *v*/*v*) for 10 min. Aliquots of pepper extract (9 μL) were mixed with 109 μL of Folin–Ciocalteau reagent, and the results were expressed as milligrams of gallic acid equivalent (GAE) per 100 g FW.

The flavonoid content was determined with quercetin as standard, using the method described previously [12], with 12.5 μL of pepper extract and 7.5 μL of 5% NaNO_2_. Aliquots of 15 μL of 10% AlCl_3_ were added after 5 min incubation, and thereafter 50 μL of 1 M NaOH was added after 6 min. The absorbance was read at 510 nm (Microplate Reader, SpectrostarNano, BMG-LABTEC, Ortenberg, Germany). Flavonoid content was calculated using a standard curve of quercetin and expressed as grams of quercetin equivalents per kilogram FW.

#### 2.4.5. Antioxidant Activity

The ferric reducing antioxidant potential (FRAP) assay was performed without modifications [15]. A fresh sample (5 g) was homogenized with methanol:water (4:1 *v*/*v*). A FRAP solution (0.3 mM sodium acetate, 10 mM 2,4,6-tripyridyl-s-triazine (TPTZ), and 20 mM FeCl_3_ (10:1:1 *v*/*v*/*v*) (950 μL; pH 3.6) at 37 °C was mixed with 50 μL of the sample mixture. A calibration curve for quantification of FRAP antioxidant activity was constructed using Trolox solution (10–250 mg L^−1^), and results were expressed as kilomoles of Trolox equivalent antioxidant capacity (TEAC) per kilogram FW.

#### 2.4.6. Phenylalanine Ammonia Lyase (PAL)

Borate buffer (150 mM, pH 8.8) of 150 µL containing 5 mM β-mercapto-ethanol and 2 mM Ethylenediamine tetraacetic acid (EDTA) were for used to determine the PAL [16]. The enzyme extract (75 mL) was incubated with 150 mL of borate buffer at 50 mM, pH 8.8, in the presence of 20 mM phenylalanine for 60 min at 37 °C. After completion of the incubation, 75 mL of 1 M HCl was added in order to stop the reaction. The final cinnamate production was determined at 290 nm using a microplate reader (SpectrostarNano BMG-LABTEC, Ortenberg, Germany). The PAL enzyme activity was expressed as nmol kg^−1^s^−1^.

### 2.5. Statistical Analysis

LED lights were arranged on shelves at 7 °C in a randomized, complete block design. The research was conducted separately for each fresh-cut sweet pepper cultivar with 10 replicates stored under each LED light treatment, including the controls, and storage time because of the seasonal availability of the sweet peppers. The data were subjected to two-way analysis of variance (ANOVA) to see the interaction effect of two independent factors (storage time and LED treatments) by a generalized linear model with the Statistical Analysis System (SAS) software program version 9.0 (SAS Institute Inc., Cary, NC, USA). Means of LED light treatments and the controls on storage time were separated by Least Significant Difference (LSD 5%). All experiments were repeated thrice.

## 3. Results

### 3.1. Commercial Value and Overall *Marketability*

**Figure 1:**
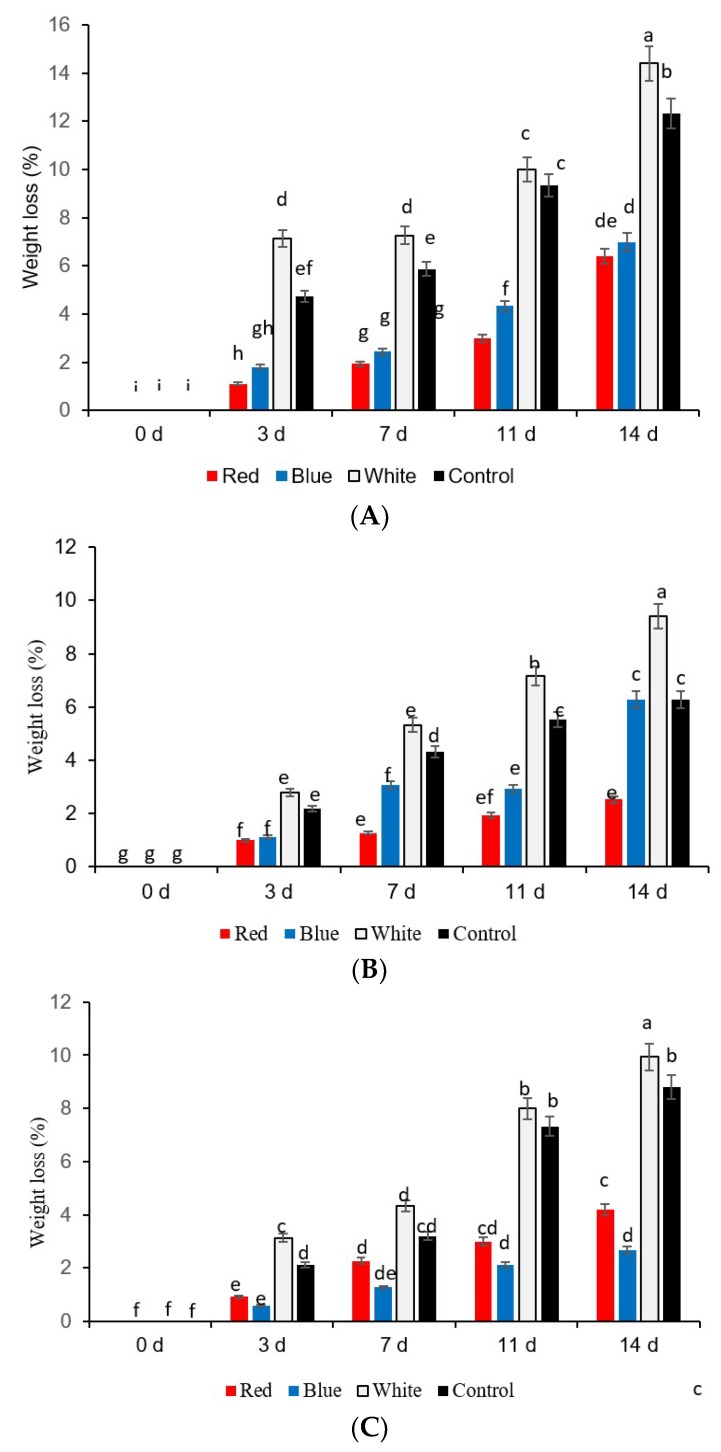
Effect of light-emitting diode (LED) treatments on weight loss of (*A*) yellow, (*B*) green, and (*C*) red sweet pepper fresh-cuts. R—Red, B—Blue, W—White light; Control—Darkness. Data include 0, 3, 7, 11, and 14 days storage time. Different letters above each bar indicate significant differences (*p* < 0.05) using Fisher’s Least Significant Difference (LSD) test.

The weight losses of fresh-cut yellow, green, and red sweet peppers under different LED lights for 8 h exposure per 24 h, related to storage periods, are expressed in percentages (Figure 1A–C). The weight loss increased significantly for three types of fresh-cut sweet peppers during 14 days exposure to white light at 7 °C (Figure 1A–C)

Yellow sweet pepper fresh-cuts exposed to white light and darkness (control) revealed significantly higher weight loss compared to those exposed to blue and red LED light from 3 to 14 day (Figure 1A). In green sweet pepper fresh-cuts, blue and red LED light treatments significantly reduced weight loss up to day 11 compared to white light and darkness (control) (Figure 1B). However, the red LED lights kept the weight loss almost to 3% in green sweet pepper fresh-cuts on day 14. In red sweet pepper fresh-cuts, the blue and red LED lights significantly reduced weight loss after 3, 7, and 14 days compared to those exposed to white light and stored in darkness (control) (Figure 1C). Therefore, it can be concluded that commercially acceptable weight loss (≤2.0%) was maintained for fresh-cuts of yellow and green sweet pepper up to days 7 and 11, with 8 h exposure with red LED light, respectively (Figure 1A,B). In red fresh-cut sweet peppers, blue LED light to for 8 h helped to reach the commercially acceptable weight loss (≤2.0%) up to day 11 (Figure 1C).

**Figure 2:**
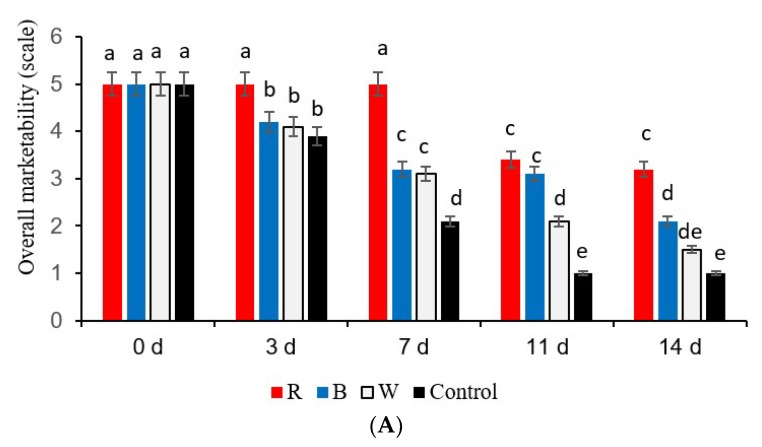

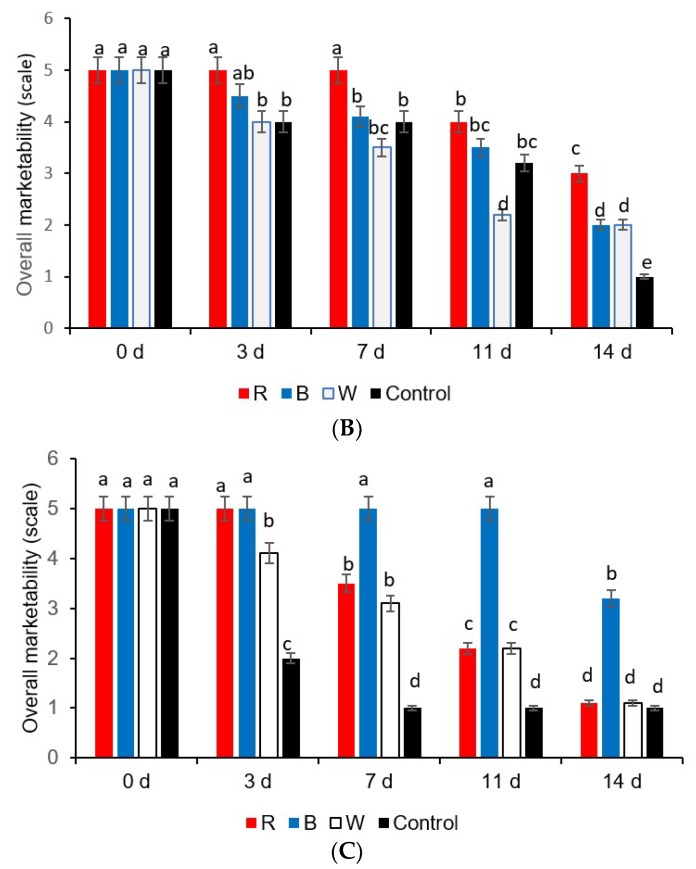
Overall marketability of (**A**) yellow, (**B**) green, and (**C**) red sweet pepper fresh-cuts exposed to LED light treatments. Where 5 = highly marketable and 0 = unmarketable. R—Red, B—Blue, W—White light; Control—Darkness. Data include 0, 3, 7, 11, and 14 days storage time. Different letters above each bar indicate significant differences (*p* < 0.05) using Fisher’s LSD test.

Marketability of the three different types of fresh-cut sweet peppers was affected differently by the LED lights (Figure 2A–C). Marketability was determined based on the browning observed on the three types of fresh-cut sweet peppers. The best marketability (scale 5) was obtained in yellow sweet pepper fresh-cuts exposed to red LED light up to day 7 (Figure 2A), and these fresh-cut sweet peppers showed absence of browning similar to day 0. Exposure to blue LED and white lights were of average quality (scale 3) with limited marketability as there was 25% browning on the 7th day, and those stored in darkness (control) showed 50% browning and became unmarketable (scale 2) (Figure 2A). The red LED lights retained the highest marketability (scale 5) of green sweet pepper fresh-cuts up to 7 days without any browning and are most suitable for the supermarkets that meet stringent quality standards. But those exposed to blue LED light and stored in darkness (control) showed 5%–10% browning and were regarded as still marketable (scale 2) at the urban fresh produce markets. The green sweet pepper samples exposed to red LED light were still marketable but showed 5%–10% browning (scale 2) on the 11th day. On the 14th day, the marketability was limited as there was 25% browning (scale 3) (Figure 2B). Samples exposed to blue LED light and white light showed limited marketability on the 7th day (scale 3) and became of unmarketable quality with 50% browning (scale 2) on the 11th day. In contrast, the samples stored in darkness (control) showed limited marketability (scale 3) on the 11th day and became unmarketable (scale 1) because there was 100% browning on day 14 (Figure 2B). On the other hand, red sweet pepper fresh-cuts exposed to blue LED light showed the highest marketability (scale 1) because there was an absence of browning up to the 11th day, similar to day 0 samples (Figure 2C). However, on day 14, the fresh-cuts became unmarketable (scale 3) because there was 25% browning. All samples exposed to red LED lights remained marketable without browning up to the 11th day (Figure 2C).

**Figure 3:**
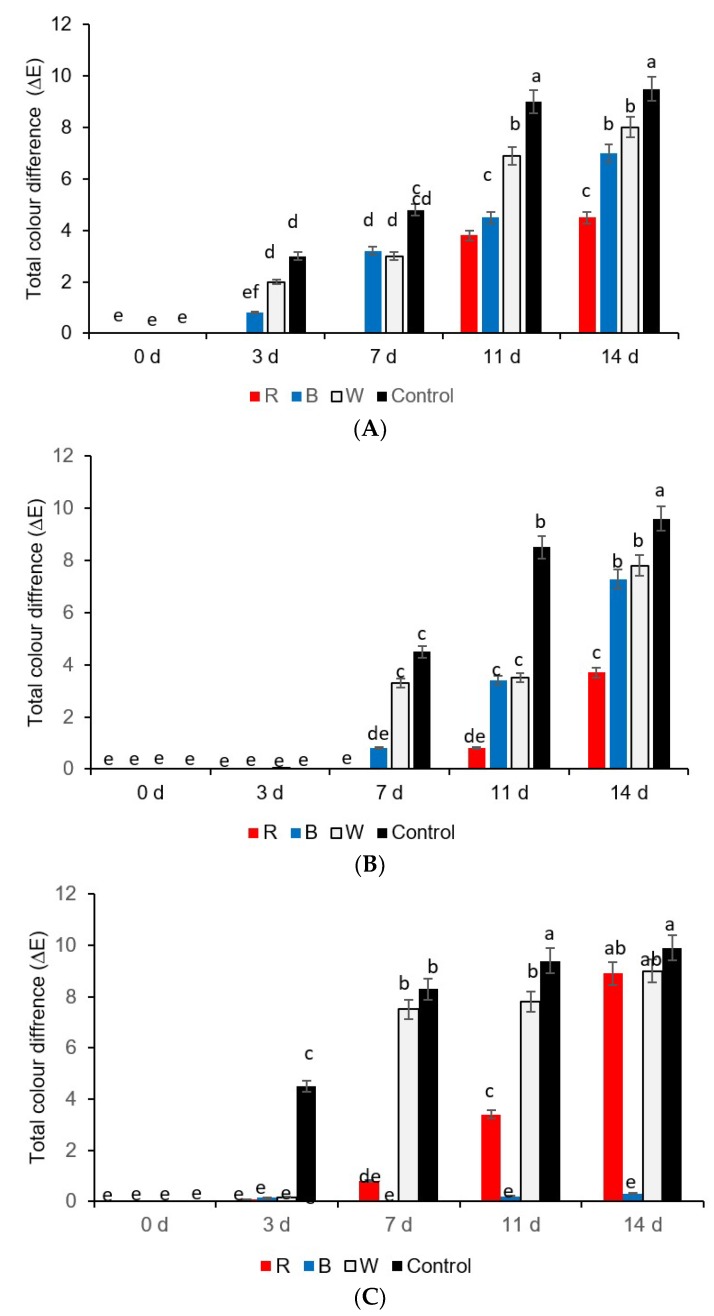
Total color changes in (**A**) yellow, (**B**) green, and (**C**) red sweet pepper fresh-cuts exposed to LED treatments. R—Red, B—Blue, W—White light; Control—Darkness. Data include 0, 3, 7, 11, and 14 days storage time. Different letters above each bar indicate significant differences (*p* < 0.05) using Fisher’s LSD test.

The total color difference (∆E) was kept to a minimum, almost similar to day 0, in yellow and green sweet pepper fresh-cuts exposed to red LED light on the 3rd and 7th days, respectively, especially because of the reduced browning (Figure 3A). Although, on the 11th day, the total color difference (∆E) significantly increased in green sweet pepper fresh-cuts, mainly because of the observed browning (scale 2), and the total color difference (∆E) was significantly lower on day 14 (Figure 3B).

Essentially, both red and blue LED treatments adopted in this study kept the total color change (∆E) to a minimum and maintained the original appearance as day 0 in red sweet pepper fresh-cuts on the 3rd day (Figure 3C). On days 3, 7, 11, and 14, the samples stored under blue LED light showed a minimum total color change (∆E) more or less similar to day 0 samples (Figure 3C). The red LED lights showed an increase in the total color change (∆E) mainly because of the observed browning (scale 3) on day 7 (Figure 3C). But those samples held under the red LED lights significantly reduced the color difference (∆E) on day 11 because there was an absence of browning (scale 5) (Figure 3C).

### 3.2. β-Carotene, Chlorophyll, and Lycopene

Yellow sweet pepper fresh-cuts exposed to red LED light retained their initial β-carotene levels up to the 7th day. Thereafter, the readings declined significantly on the 11th and 14th days (Table 1). It is noteworthy that on the 11th and 14th day, 10.37%–14.62% of β-carotene was lost in samples exposed to red LED light, which was significantly lower than samples stored in the dark (control), showing losses around 35.37% and 61.32% on the 11th and 14th day, respectively (Table 1). However, samples exposed to blue LED and white lights already lost around 17.45% and 25.4% of β-carotene by the 7th day, respectively (Table 1).

All treatments adopted in this study helped to retain the total chlorophyll content in green sweet pepper fresh-cuts on the 3rd day. However, samples exposed to red LED light on 7 days storage clearly retained the highest concentration of chlorophyll, similar to the concentrations observed on day 0 (Table 1). On the 7th day, samples exposed to blue LED light, white light, and held in darkness (control) lost around 15.36%, 23.82%, and 45.14% of chlorophyll content, respectively (Table 1).

The lycopene content of the fresh-cut red sweet peppers was maintained at similar concentrations as day 0 samples on day 3 in all treatments other than the control (darkness) (Table 1). However, from day 7 up to day 14, the red LED light showed optimal retention of lycopene content at levels similar to day 0 in red sweet pepper fresh-cuts (Table 1). Blue LED light also favored retention of lycopene, similar to red LED light on the 7th day. However, the red LED light clearly improved retention of lycopene on the 11th and 14th day compared to blue LED or white light and the control (darkness). On the 11th and 14th day, the lowest lycopene concentration was observed in the control samples (darkness) (Table 1). Almost 40% and 42% of lycopene was lost in red sweet pepper fresh-cuts stored in white light and darkness, respectively, on the 14th day (Table 1).

### 3.3. Ascorbic Acid, Total Phenols, Flavonoids, and Antioxidant Activity

Yellow and green fresh-cut samples exposed to red LED light showed the highest concentration of ascorbic acid on days 3 and 7, with concentrations similar to those detected in samples from day 0 (Table 2). Ascorbic acid content in the yellow sweet pepper fresh-cut samples stored in darkness (control) decreased significantly and showed the lowest levels on the 11th and 14th days (Table 2). The concentration of ascorbic acid in fresh-cut green sweet pepper samples exposed to red LED light declined significantly after the 7th day onwards. Also, the ascorbic acid content in fresh-cut green sweet pepper samples exposed to blue LED light, white light, and held in darkness (control) declined by day 7. The ascorbic acid concentrations in fresh-cut green sweet pepper samples exposed to blue LED light, white light, and held in darkness (control) did not vary significantly on days 11 and 14 (Table 2). In red sweet pepper fresh-cuts, blue LED light helped to retain the ascorbic acid content up to day 11. Samples exposed to white light on day 14 and held in darkness (control) on days 11 and 12 showed lower concentrations of ascorbic acid (Table 2).

At the same time, it is interesting to note that 16.45%, 20.25%, and 26.5% of ascorbic acid was lost on day 7 in fresh-cut yellow sweet peppers exposed to blue LED light, white light, and those held in darkness (control), respectively (Table 2). In green sweet pepper fresh-cuts, 27.27%, 32.32%, and 33.83% of ascorbic acid was lost on day 7 when exposed to blue LED light or white light or held in darkness (control), respectively (Table 2). Similarly, in red sweet pepper fresh-cut samples exposed to red LED light, white light, and those held in darkness (control) lost 14.28%, 29.71%, and 44.57% of ascorbic acid, respectively (Table 2).

Total phenolic content increased significantly to the highest level on the 7th day in yellow sweet pepper samples exposed to red LED light (Table 2). But in green fresh-cut sweet peppers, the highest concentration of total phenolic compounds was obtained in samples exposed to red LED light on days 3 and 7 during storage. In red sweet peppers, the blue LED light exposure caused a significant increase in total phenols from the 3rd to the 11th day (Table 2).

In yellow sweet pepper fresh-cut samples exposed to blue LED and white light, the concentrations of total phenolics were significantly lower than those exposed to red LED light on day 11 (Table 2). However, on day 14, samples exposed to white light and held in darkness for 14 days showed significantly lower concentrations of phenolic content than the yellow sweet pepper fresh-cuts exposed to blue or red LED lights (Table 2). It is noteworthy that in green sweet pepper fresh-cuts held in darkness (control) for 11 and 14 days and red sweet pepper fresh-cuts stored under similar conditions for 14 days showed significantly lower concentrations of total phenols (Table 2).

Similar observations were noted in red sweet pepper fresh-cuts exposed to blue LED light on the 7th and the 11th day (Table 2). At the same time, the concentration of total phenols in yellow and green sweet pepper fresh-cuts samples stored in darkness (control) for 3 days showed similar concentrations as those samples from day 0. Thereafter, on day 7, the total phenol concentration declined, and a nonsignificant difference in concentration was observed in those samples on day 7 and 11. All three types of pepper samples stored in darkness (control) showed moderately lower concentrations of total phenolic content on the 7th day (Table 2).

The flavonoid quercetin content showed the highest concentration on the 3rd and 7th day in yellow sweet pepper fresh-cuts exposed to red LED light (Table 2). However, in green sweet pepper fresh-cuts exposed to red LED light , the highest concentration of quercetin content was noted on the 7th day. In red sweet pepper fresh-cuts, the quercetin content was significantly highest on the 7th and 11th day under blue LED light (Table 2). It must be noted that the total phenols and flavonoid quercetin concentration were lower in all three types of sweet pepper fresh-cuts on day 0. Unlike in yellow and green sweet pepper fresh-cuts, the red sweet pepper fresh-cuts held in the darkness (control) for 0 to 3 days showed significantly higher concentrations of quercetin content compared to the 11 and 14 days samples (Table 2).

**Figure 4:**
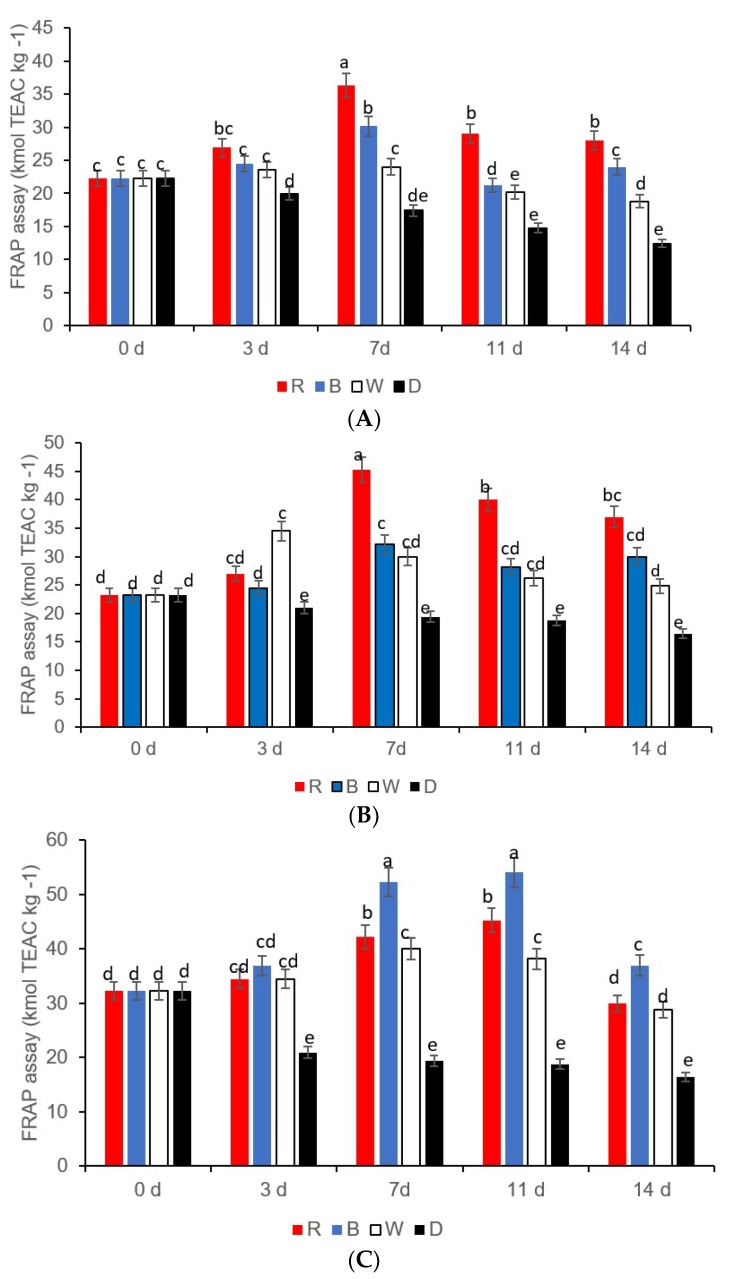
Antioxidant activity in (**A**) green sweet pepper, (**B**) yellow sweet pepper, and (**C**) red sweet pepper fresh-cuts exposed to different LED light treatments for 8 h at 7 °C. R—Red, B—blue, W—white light; Control—Darkness. Data include 0, 3, 7, 11, and 14 days storage time. Different letters above each bar indicate significant differences (*p* < 0.05) using Fisher’s LSD test.

The total antioxidant capacity showed a significant increase in yellow and green sweet peppers exposed to red LED light on the 7th day (Figure 4A–B), whilst in red sweet pepper fresh-cuts, the total antioxidant capacity was highest on days 7 and 14 in samples exposed to blue LED light (Figure 4C). The yellow sweet pepper samples stored in darkness (control) showed the lowest antioxidant capacity compared to all the light treatments from the 7th day onwards, but in green and red sweet pepper fresh-cuts, after day 3 there was no significant difference in antioxidant capacity observed (Figure 4A–C).

### 3.4. PAL Activity

**Figure 5:**
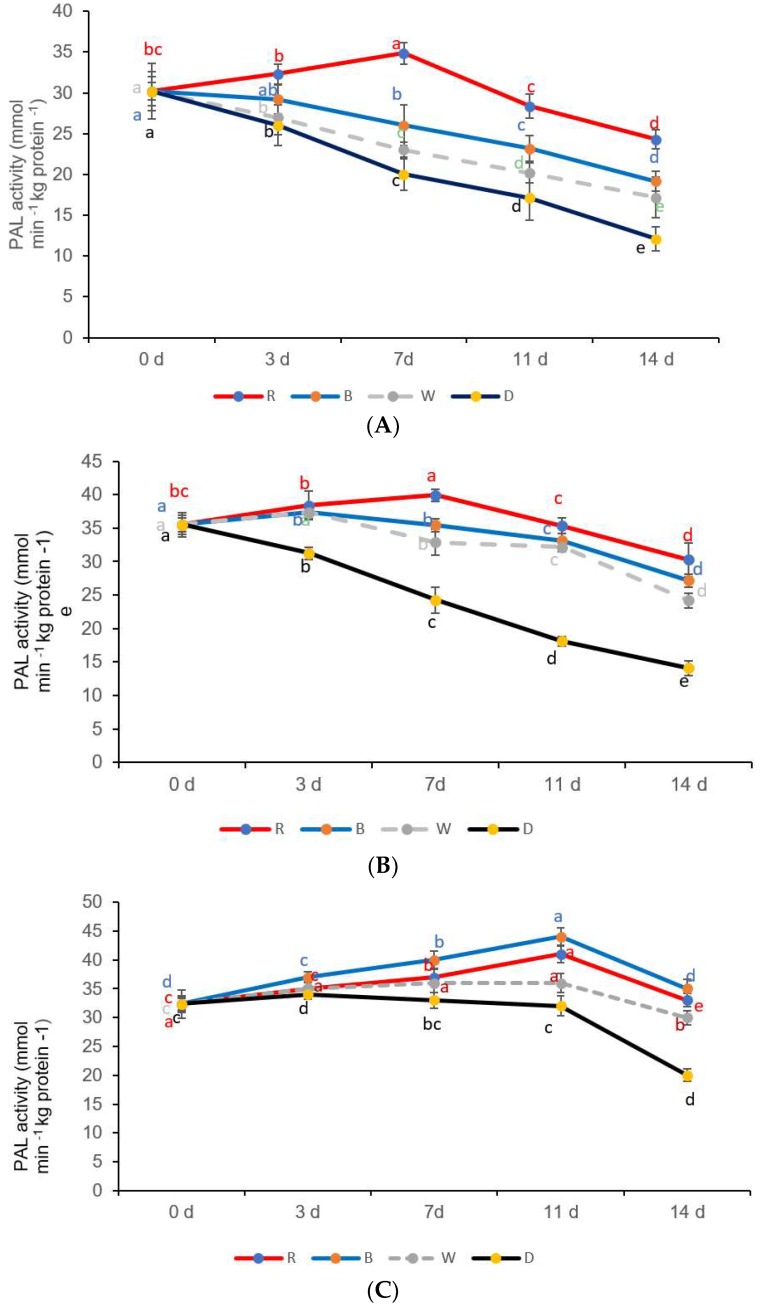
Phenylalanine ammonia lyase (PAL) activity in (**A**) yellow sweet pepper, (B) green sweet pepper, and (**C**) red sweet pepper fresh-cuts exposed to different LED light treatments and storage times from 0 to 14 days for 8 h at 7 °C. R —Red, B—Blue, W—White light; D—Darkness (control). Data include 0, 3, 7, 11, and 14 days storage time. Different letters above each bar indicate significant differences (*p* < 0.05) using Fisher’s LSD test.

The PAL activity in the yellow and green peppers exposed to red LED light was highest on the 7th day and declined significantly afterwards (Figure 5A,B). However, a different trend regarding PAL activity was noted in red peppers exposed to blue LED light, showing a gradual increase, and on day 11, a significant increase in activity was observed with a subsequent decline thereafter (Figure 5C). A gradual decline in PAL activity was visible in all sweet pepper fresh-cuts stored in the dark (control) (Figure 5A–C). The decline in PAL activity was significant after the 3rd day in yellow and green sweet peppers exposed to red LED light (Figure 5A,B). In red sweet pepper fresh-cuts, the PAL activity increased significantly on the 3rd day, remained stable, and thereafter declined significantly on the 7th day (Figure 5C).

## 4. Discussion

It is evident from this study that LED light treatments can be employed in a storage system to extend the shelf life of fresh-cut sweet peppers by controlling physiological processes and biochemical reactions. Weight loss is referred to as moisture loss generally associated with the respiration of fresh-cut produce; hence, the implemented breakpoint for maintaining the quality of fresh produce is critical when the moisture content drops by 5% [17]. The difference in weight loss observed in darkness (control) among the different types of sweet peppers could be attributed to the differences in varying thicknesses of epidermal and hypodermal layers [18]. In addition, in this study, the fresh-cut sweet peppers were packaged in commercial cling film in tray packs, and continuous white light could have probably caused an increase in moisture loss due to an increased rate of respiration rather than the effect of the light on the stomatal openings observed in leafy vegetables such as broccoli [19]. The red LED light reduced weight loss, probably because of the reduced rate of respiration, and prevented water loss during respiration in the fresh-cut sweet peppers. However, respiration needs to be determined in this study to prove the impact of LED lights on the rate of respiration in fresh-cut peppers. Since the red and blue LED lights played a major role in reducing the weight loss and the color differences in fresh cuts of green or yellow and red sweet peppers, respectively, red and blue LED lights are beneficial in extending the shelf life of the fresh cuts of green or yellow and red sweet peppers at the retail shelf or during storage.

The effect of LED lights on the rate of respiration in banana was reported previously [20]. Bananas exposed to red LED light reduced the rate of respiration than those exposed to blue LED, especially after 11 days [20]. Furthermore, several studies indicated that the LED lights delayed senescence of broccoli [21] better than the samples kept in the dark.

The multiple photoreception system (chlorophylls, carotenoids, phytochrome, cryptochrome, phototropins) in a plant organ, the quality of the selected LED lights, and the exposure time all play a role in determining the degree of influence of selected LED lights on physiological processes and biochemical reactions [20,21]. Furthermore, the postharvest response of different light spectra can vary within cultivars [12]. Higher ratios of R:FR spectral lights increased the lycopene content in tomatoes [3]. In contrast, moderate (red light) R:FR (far red light) ratios of spectrum lights were shown to increase the lycopene content in sweet peppers [12]. However, in this study, the exposure to red LED light increased the lycopene content on the 11th day in red fresh-cut sweet peppers (Figure 5A). Phytochrome facilitated red light induced carotenoid and lycopene synthesis in tomatoes [22]. Red LED lights induced the gene expression and carotenoid synthesis in the flavedo of Satsuma mandarin (*Citrus unshiu* Marc.) [21]. Similarly, the biosynthesis of β-carotene in yellow sweet pepper fresh-cuts induced by red LED lights up to 7 days (Figure 4B) could possibly be due to similar inductions of gene expression in the carotenoid synthesis pathway, but detailed investigations to prove this argument need to be carried out in the future. Although carotenoid synthesis is reported to occur under light conditions, photo-oxidation could destroy them [23], which could probably be the reason for the lower β-carotene and lycopene contents on day 14 in the fresh-cut yellow sweet peppers exposed to white light in this study (Figure 5B). Red and blue light were reported to improve the chlorophyll content in plant leaves, but red-light exposure was reported previously to delay the degradation of the chloroplast ultrastructure [24]. This could be the reason for the observed higher chlorophyll content noted in the fresh-cut green sweet peppers on day seven under red LED light (Figure 5B).

Exposure to blue/red LED lights induced secondary metabolites (phenols and flavonoids) via phenylalanine ammonia-lyase enzyme (PAL), the primary step of the phenyl propanoid pathway (Hao et al., 2003). The phytochrome photoreceptors that have two interconvertible forms, the inactive Pr and the active Pfr, have a sensitivity peak in the red (564–580 nm) [25] and blue (440–450 nm) light spectrum absorbed by the cryptochrome and phototropin photoreceptors [26,27]. Therefore, the blue or red LED lights possibly could have activated the phytochrome system in yellow and green sweet peppers (until day 7), and cryptochrome and phototropin in red sweet pepper (until day 11), which promote the concentration of sugars (soluble carbohydrates), the precursors for the biosynthesis of ascorbic acid, or polyphenolic compounds, such as phenolic acids, thereby improving the visual quality (chlorophyll, lycopene, and carotenoids) and extending the shelf life. However, an in-depth investigation is needed to understand the mechanisms. Antioxidant properties of pea [28,29] and cabbage [17] have been improved by LED red/blue light compared to white light. Therefore, LED lights (red or blue) in the storage system improved the nutritional quality of the fresh-cut sweet peppers to the benefit of consumers. At the same time, increased biosynthesis of phenolics under red LED (green and yellow sweet pepper fresh-cuts) and blue LED light (red sweet pepper fresh-cuts) improved antioxidant properties and extended the shelf life by delaying senescence of the yellow and red sweet peppers, since antioxidants participate in scavenging the free radicals formed during the metabolic pathways [30].

Broccoli heads exposed to blue LED light during postharvest storage showed lower ascorbic acid content compared to red light, and a similar observation was noted in this study [21]. Higher light irradiance levels stimulated the biosynthetic pathway of ascorbic acid biosynthesis [31].

## 5. Conclusions

Application of red LED lights for 8 h per day during storage at 7 °C was beneficial to retain the commercial quality, bioactive compounds, and antioxidant activity as well as extend the shelf life of fresh-cut yellow and green sweet pepper up to 7 d. Exposure to blue LED light can be recommended for fresh-cut red sweet peppers up to 11 days. Thus, the use of suitable LED light in the postharvest environment could be a novel approach for the fresh-cut industry to reduce postharvest losses of fresh-cut sweet peppers at the retail shelf.

## Figures and Tables

**Table 1:** Influence of red or blue LED lights and storage times from 0 to 14 days on β-carotene content in yellow sweet pepper, chlorophyll content in green sweet pepper, and lycopene content in red sweet pepper fresh-cuts at 7 °C.

LED Lights and Storage Time (d)	β-Carotene (g kg^−1^)	Chlorophyll (g kg^−1^)	Lycopene (g kg^−1^)
Red LED light x 0 day	0.212 a ± 0.01	3.19 a ± 0.02	0.165 a ± 0.03
Red LED light x 3 days	0.213 a ± 0.01	3.19 a ± 0.05	0.167 a ± 0.06
Red LED light x 7 days	0.214 a ± 0.02	3.00 a ± 0.03	0.168 a ± 0.01
Red LED light x 11 days	0.190 b ± 0.01	2.7 b ± 0.04	0.166 a ± 0.02
Red LED light x 14 days	0.181 b ± 0.04	1.75 c ± 0.08	0.160 ab ± 0.03
Blue LED light x 0 days	0.212 a ± 0.01	3.19 a ± 0.01	0.165 a ± 0.02
Blue LED light x 3 days	0.187 b ± 0.05	3.12 a ± 0.05	0.165 a ± 0.01
Blue LED light x 7 days	0.175 b ± 0.10	2.70 b ± 0.02	0.162 a ± 0.02
Blue LED light x 11 days	0.143 c ± 0.08	2.70 b ± 0.02	0.140 b ± 0.10
Blue LED light x 14 days	0.141 c ± 0.03	1.87 c ± 0.10	0.138 b ± 0.08
White light x 0 day	0.212 a ± 0.02	3.19 a ± 0.02	0.165 a ± 0.01
White light x 3 days	0.175 b ± 0.06	3.07 a ± 0.07	0.165 a ± 0.07
White light x 7 days	0.158 b ± 0.03	2.43 b ± 0.01	0.120 b ± 0.12c
White light x 11 days	0.141 c ± 0.02	1.86 c ± 0.04	0.115 c ± 0.07
White light x 14 days	0.089 d ± 0.02	1.70 c ± 0.08	0.114 c ± 0.05
Darkness x 0 days	0.212 a ± 0.02	3.19 a ± 0.01	0.165 a ± 0.03
Darkness x 3 days	0.184 b ± 0.03	3.07 a ± 0.06	0.113 c ± 0.14
Darkness x 7 days	0.140 c ± 0.07	1.75 c ± 0.05	0.106 cd ± 0.05
Darkness x 11 days	0.137 c ± 0.04	1.74 c ± 0.03	0.099 d ± 0.02
Darkness x 14 days	0.082 d ± 0.03	1.68 c ± 0.09	0.093 d ± 0.01

Values are means ± standard deviation of ten replicates. Mean values with same alphabetic letters in a column for a specific parameter are not significantly different according to LSD tests (*p* < 0.05).

**Table 2:** Effect of LED lights and different storage times from 0 to 14 days on ascorbic acid, total phenols, and flavonoid (quercetin) concentrations in yellow, green, and red sweet pepper fresh-cuts at 7 °C.

	Fresh-Cuts								
LED Lights and Storage Time (d)	Yellow Sweet Pepper			Green Sweet Pepper			Red Sweet Pepper		
	Ascorbic Acid Content (g kg^−1^)	Total Phenols (g kg^−1^)	Total Flavonoid (Quercetin Content) (g kg^−1^)	Ascorbic Acid Content (g kg^−1^)	Total Phenols (g kg^−1^)	Total Flavonoid (Quercetin Content) (g kg^−1^)	Ascorbic Acid Content (g kg^−1^)	Total Phenols (g kg^−1^)	Total Flavonoid (Quercetin Content) (g kg^–1^)]
Red LED light x 0 day	1.58 a+0.03	0.96 bc ± 0.09	0.028 c ± 0.03	1.98 a ± 0.06	0.72 bc ± 0.02	0.020 c ± 0.02	1.75 a ± 0.05	1.02 c ± 0.04	0.034 c ± 0.09
Red LED light x 3 days	1.57 a ± 0.09	1.01 b ± 0.03	0.043 a ± 0.12	1.96 a ± 0.04	1.02 a ± 0.04	0.030 b ± 0.06	1.73 a ± 0.02	1.26 b ± 0.09	0.036 c ± 0.02
Red LED light x 7 days	1.56 a ± 0.04	1.28 a ± 0.05	0.045 a ± 0.15	1.95 a ± 0.19	1.07 a ± 0.18	0.035 a ± 0.03	1.72 a ± 0.09	1.29 b ± 0.03	0.042 bc ± 0.11
Red LED light x 11 days	1.34 b ± 0.12	1.10 b ± 0.01	0.035 b ± 0.09	1.70 b ± 0.02	0.80 b ± 0.12	0.027 b ± 0.05	1.50 b ± 0.13	1.30 b ± 0.01	0.038 c ± 0.07
Red LED light x 14 days	1.26 bc ± 0.04	0.87 bc ± 0.11	0.032 bc ± 0.06	1.45 c ± 0.12	0.78 bc ± 0.02	0.029 b ± 0.03	1.23 c ± 0.01	1.01 c ± 0.21	0.035 c ± 0.14
Blue LED light x 0 days	1.58 a ± 0.03	0.96 bc ± 0.09	0.028 c ± 0.04	1.98 a ± 0.6	0.76 bc ± 0.02	0.021 c ± 0.04	1.75 a ± 0.05	1.02 c ± 0.04	0.034 c ± 0.01
Blue LED light x 3 days	1.33 b ± 0.15	0.94 bc ± 0.17	0.032 bc ± 0.25	1.94 a ± 0.16	0.98 ab ± 0.04	0.025 bc ± 0.01	1.73 a ± 0.19	1.55 a ± 0.010	0.050 b ± 0.05
Blue LED light x 7 days	1.32 b ± 0.02	0.84 bc ± 0.03	0.034 bc ± 0.02	1.69 b ± 0.08	0.80 b ± 0.15	0.028 b ± 0.09	1.75 a ± 0.12	1.60 a ± 0.08	0.064 a ± 0.12
Blue LED light x 11 days	1.21 c ± 0.13	0.67 c ± 0.12	0.035 b ± 0.04	1.44 c ± 0.01	0.79 b ± 0.16	0.027 b ± 0.01	1.70 a ± 0.14	1.63 a ± 0.04	0.067 a ± 0.16
Blue LED light x 14 days	1.20 c ± 0.095	0.56 cd ± 0.06	0.032 bc ± 0.01	1.30 cd ± 02	0.78 b ± 0.07	0.026 b ± 0.04	1.45 b ± 0.09	1.30 b ± 0.02	0.049 b ± 0.02
White light x 0 days	1.58 a ± 0.03	0.96 bc ± 0.09	0.028 c ± 0.01	1.98 a ± 0.06	0.72 bc ± 0.02	0.020 c ± 0.07	1.75 a ± 0.05	1.02 c ± 0.04	0.034 c ± 09
White light x 3 days	1.31 b ± 0.08	0.91 a ± 0.06	0.030 c ± 0.03	1.95 a ± 0.04	0.73 bc ± 0.03	0.021 c ± 0.02	1.72 a ± 0.18	1.24 b ± 0.08	0.033 c ± 0.14
White light x 7 days	1.26 c ± 0.02	0.75 c ± 0.11	0.030 c ± 0.04	1.43 c ± 0.08	0.74 bc ± 0.15	0.025 bc ± 0.06	1.24 c ± 0.12	1.26 b ± 0.01	0.034 c ± 0.09
White light x 11 days	1.20 c ± 0.01	0.52 d ± 0.05	0.030 c ± 0.17	1.38 cd ± 0.15	0.72 bc ± 0.08	0.025 bc ± 0.03	1.23 c ± 0.07	1.29 b ± 0.21	0.033 c ± 0.12
White light x 14 days	1.17 c ± 0.12	0.50 d ± 0.13	0.030 c ± 0.03	1.18 d ± 0.04	0.70 bc ± 0.02	0.021 c ± 0.08	1.10 cd ± 0.04	0.98 c ± 0.13	0.032 c ± 0.17
Darkness x 0 days	1.58 a ± 0.03	1.30 cd ± 02	0.028 c ± 0.02	1.98 a ± 0.06	0.72 bc ± 0.02	0.020 c ± 0.11	1.75 a ± 0.05	1.02 c ± 0.04	0.034 c ± 09
Darkness x 3 days	1.32 b ± 0.09	0.84 bc ± 0.02	0.029 c ± 0.19	1.92 a ± 0.03	0.65 c ± 0.03	0.020 c ± 0.03	1.23 c ± 0.10	0.98 c ± 0.17	0.032 c ± 0.01
Darkness x 7 days	1.16 c ± 0.15	0.52 d ± 0.17	0.030 c ± 0.03	1.31 cd ± 0.18	0.64 c ± 0.18	0.021 c ± 0.07	1.11 cd ± 0.17	0.96 c ± 0.04	0.030 cd ± 0.15
Darkness x 11 days	0.81 d ± 0.06	0.50 d ± 0.12	0.030 c ± 0.12	1.30 cd ± 0.01	0.42 d ± 0.04	0.025 bc ± 0.04	0.96 d ± 0.02	0.96 c ± 0.02	0.021 d ± 0.06
Darkness x 14 days	0.78 d ± 0.02	0.29 e ± 0.01	0.029 c ± 0.04	1.14 d ± 0.12	0.41 d ± 0.01	0.021 c ± 0.02	0.97 d ± 0.12	0.71 d ± 0.15	0.020 d ± 0.08

Values are mean ± standard deviation of ten replicates. Mean values with same alphabetic letters in a column for a specific parameter are not significantly different according to LSD tests (*p* < 0.05).
